# Determination of risk factors influencing substance use among Turkish-origin immigrants in Germany

**DOI:** 10.55730/1300-0144.5893

**Published:** 2024-07-04

**Authors:** Enes EFENDİOĞLU, Lütfiye Hilal ÖZCEBE

**Affiliations:** 1 Department of Public Health, Graduate School of Health Sciences, Hacettepe University, Ankara, Turkiye; 2 Department of Public Health, Faculty of Medicine, Hacettepe University, Ankara, Turkiye

**Keywords:** Substance use, risk factors, Turkish immigrants, Germany, intergenerational analysis, tobacco

## Abstract

**Background/aim:**

Increasing international migration poses unique challenges, especially regarding health outcomes and behaviors such as substance use. This study aims to identify risk factors influencing substance use among Turkish-origin immigrants in Germany.

**Materials and methods:**

Data were obtained from the STEPS survey conducted by the Presidency for Turks Abroad and Related Communities (YTB) in 2021 in Germany. A total of 1157 participants were selected through a quota sampling method. Data analysis involved descriptive statistics and logistic regression utilizing IBM SPSS Statistics 21.

**Results:**

The study found a significant association between substance use and variables such as generation status, age, and sex. Third-generation immigrants showed a higher propensity for substance use compared to the first and second generations. Significant relationships were also observed between substance use and other risk behaviors such as tobacco and alcohol consumption.

**Conclusion:**

Substance use among Turkish-origin immigrants in Germany is influenced by generational status, with younger and third-generation individuals being at higher risk. Additionally, tobacco and alcohol use are strong predictors of substance use, highlighting the need for targeted interventions, especially for young migrants.

## 1. Introduction

Europe holds a prominent position among the regions receiving the most immigration worldwide. The social, economic, and legal rights offered by European countries make these nations particularly appealing in the eyes of citizens from other countries. Among European nations, Germany has been a significant “hosting country” for decades [1]. According to data published by the German Federal Statistical Office, one in five people in Germany has an immigration background, meaning that either they moved to Germany themselves or at least one of their parents migrated from a different country to Germany.^1^

Following World War II, West European states, led by the German government, faced an increased need for human resources due to poverty and deaths resulting from the war [2]. Consequently, labor migration from various countries, including Türkiye, was encouraged to West European countries, particularly Germany. Official Turkish labor migration began with the “Turkish-German Worker Exchange Agreement” signed between Türkiye and Germany on 31 October 1961 [3]. Following this labor agreement, approximately 600,000 individuals migrated to Germany as “guest workers” (*Gastarbeiter*) [4]. Currently, immigrants of Turkish origin constitute 18.4% of the migrant population in Germany.^2^

Even decades after migration, immigrant-origin populations face social integration stress, sometimes experiencing unequal access to basic health services compared to the host society, occupational hazards, and work stress due to the nature of their jobs. These factors contribute to differing health conditions and levels of well-being compared to the host society [5]. Immigrants’ experiences in their countries of origin, migration routes, entry into host countries, and integration policies in those countries, along with their living and working conditions, create a wide range of physical and mental health needs. These experiences can increase the vulnerability of refugees and immigrants to various health problems.^3^

The literature examining the relationship between the duration of migration and substance use has yielded varied results. A 2007 study in the United States reported that among Mexican immigrants, longer durations since migrating to the US were associated with increasingly higher rates of substance use compared to similar age groups in Mexico [6]. Thus, longer exposure to the postmigration culture implies, despite various confounding factors, higher stress and increased risk of addiction. Another study showed that immigrant groups returning to their home countries from the United States had higher rates of substance use the longer they had stayed in the US [7].

The increasing rates of substance use among immigrants are significant not only for health concerns but also for integration into the host society and in addressing social inequalities [8]. Studies have revealed that individuals using any addictive substance, including tobacco or alcohol, tend to have higher rates of substance use [9]. Substance use can affect social life and lead to health problems impacting the liver, kidneys, nervous system, and cardiovascular system, as well as adversely affecting mental health [10]. The rising rates of substance use among individuals with a migration background not only threaten these individuals but also pose a risk to society at large [11]. In this context, researching substance use among immigrant populations and understanding the factors associated with usage and behavioral patterns will guide interventions aimed at prevention. This study investigates substance use status among Turkish-origin immigrants in Germany and aims to identify the associated factors.

## 2. Materials and methods

### 2.1. Research design and sample selection

This study was conducted using data obtained from the STEPS survey administered to Turkish-origin immigrants in Germany by the Presidency for Turks Abroad and Related Communities (YTB) between June 2021 and August 2021. The data were acquired with that institution’s permission and analyzed accordingly.

The population of this research consists of Turkish-origin immigrants residing in Germany. According to data from the German Federal Statistical Office, as of 2020 there were approximately 3 million Turkish-origin immigrants in Germany, with 2.1 million aged 18 and above. Following the methods and details provided in the World Health Organization’s Stepwise Approach to Surveillance (STEPS) guide, the YTB calculated that a representative sample from this population of 2.1 million would consist of 1152 individuals; a total of 1157 participants were included in the study. These 1157 participants were distributed proportionally across each state of Germany based on the ratio of Turkish-origin immigrants to the total population of the state, and they were contacted through quota sampling.

The sample group was identified using a Turkish names database. Names from this database were randomly searched in open phone directories in Germany.

The World Health Organization’s STEPS questionnaire was administered in the study. Data collection was performed via computer-assisted telephone interviewing. Interviewers proficient in both German and Turkish took part in the research. Participants reached by phone agreed to participate in the survey and could choose to respond in either German or Turkish.

The survey form and research design applied within the scope of the study were approved by the Hacettepe University Non-Interventional Clinical Research Ethics Committee (approval date: 4 May 2021, decision number: 2021/10-53).

### 2.2. Research variables

The table of dependent and independent variables examined in the logistic regression model within the scope of this research is shown in Table 1.

In this research, the status of substance use, cigarette smoking, and alcohol consumption among participants was assessed based on whether they had ever used these substances (“ever usage”). Responses of “I have never used in my lifetime” were categorized into one group, while “I have used before” and “I am currently using” responses were combined into another group for analysis.

### 2.3. Statistical analysis

Statistical analyses were conducted using IBM SPSS Statistics 21. Summary statistics for the data included count, percentage, mean, standard deviation, and minimum and maximum values. Depending on the measurement levels of the data, chi-square analysis was used for relationship analyses and logistic regression analysis for risk assessments. A significance level of 0.05 was adopted, where p < 0.05 indicated a significant difference or relationship and p > 0.05 indicated no significant difference or relationship.

Initially, descriptive statistics for the dependent and independent variables were obtained. Chi-square tests were then conducted to determine whether there was a relationship between individuals’ substance use status and demographic factors, as well as their tobacco and alcohol use status.

In our logistic regression model, the dependent variable was binary (1- “Never used drugs,” 2- “Have used before or currently using”). Hence, a binary logistic regression model was employed. Before establishing the binary logistic regression model, the presence of multicollinearity among the independent variables was tested. Following this, binary logistic regression analysis was applied to identify the factors affecting individuals’ substance use.

## 3. Results

In this study, 1157 Turkish-origin immigrants residing in Germany were reached. It was observed that 67.5% of the 1157 participants were first-generation immigrants, 56.4% were over the age of 40, 50.8% were male, and 14.1% had parents who were relatives. Of the 846 participants who responded to the question about their total years of education, 51.3% indicated 9–15 years and 24.0% indicated ≥16 years of education. Furthermore, 41.6% of the participants were “workers/salaried employees/government officials” and 11.2% were tradespeople. The other participants did not actively work in income-generating jobs. As seen in Table 2, 43.9% of participants reported having 3 or more individuals over the age of 18 living in their households. As seen in Table 3, 45.5% of participants had smoked cigarettes at some point in their lives, 31.6% had consumed alcoholic beverages, and 14.6% had used other substances.

It was observed that among those who were born in Germany themselves and those whose parents were also born in Germany (i.e., third generation), the rate of those answering “I have used/am using” for addictive substances was significantly higher at 29.4% compared to other generations (p < 0.001). Similarly, in the age group of 18–19 years, the rate of those answering “I have used/am using” for addictive substances was significantly higher at 48.0% compared to other age groups (p < 0.001). Among male respondents, the rate of those answering “I have used/am using” for addictive substances was significantly higher at 19.6% compared to female respondents at 9.4% (p < 0.001).

Among students, the rate of those answering “I have used/am using” for addictive substances was significantly higher at 35.8% compared to other occupational groups (p < 0.001). Substance use was found to be higher among those who smoked tobacco and consumed alcoholic beverages (23.1% and 34.6%, respectively; p < 0.001) (Table 4).

In logistic regression analysis, the generation variable was a significant factor at a 95% confidence level in the established model. Upon examining the significance levels of the generation variable, it was found that individuals in the third generation were 1.37 times more likely to use substances compared to those in the second generation. Furthermore, individuals in the third generation were 2.70 times more likely to use substances than those in the first generation.

Sex did not show a significant contribution to substance use. Although not statistically significant, men were 1.12 times more likely to use substances compared to women (p > 0.05).

The number of people living in the household did not show a significant contribution to substance use. However, although not statistically significant, the rate of substance use in households with 3 or more people was 1.35 times higher compared to those with 1 or 2 people (p > 0.05).

Individuals who used tobacco were 2.55 times more likely to use other substances compared to nonusers (p < 0.05). Similarly, individuals who consumed alcohol were 6.67 times more likely to use other substances compared to those who did not consume alcohol (p < 0.05) (Table 5).

## 4. Discussion

In this study, the patterns of substance use among Turkish-origin immigrants in Germany and the risk factors influencing their usage were examined. It was found that 14.6% of the participants engaged in substance use. In a cross-sectional study conducted in 2020 by Ünübol and Hızlı Sayar in the adult population of Türkiye, it was reported that 4.5% of individuals had used substances at some point in their lives [12]. A study by İlhan et al. reported that the substance use rate was 4.3% in the Turkish population of Şanlıurfa Province and 2.6% in refugee camps in the same region [13]. The low rate of substance use reported in the immigrant population in that study could be attributed to the controlled living conditions and security/health measures in camp settings. The rate of substance use among Turkish-origin immigrants in Germany is significantly higher compared to both the general population of Türkiye and immigrants in refugee camps in Türkiye.

Previous research identified tobacco smoking and alcohol consumption as leading risk factors for substance use. A prospective study among adolescents and young adults in six European countries revealed that early use of tobacco and alcoholic beverages increased the likelihood of substance use to varying degrees [14]. Consistent with the literature, nearly half of the participants in this study were found to be smokers. These findings are in line with previous research. According to a study by Reiss et al., 40.2% of Turkish-origin immigrants in Germany smoke [15]. Donath et al. found that Turkish-origin youth consumed alcohol less frequently compared to other immigrant groups [16]. Sirin et al. stated that failure to acculturate puts immigrant youth at risk for substance use, indicating that immigrants who use substances are the ones who experience problems with acculturation [17]. High rates of tobacco smoking and alcohol consumption among the immigrant population are linked to problems in acculturation with the host society; the high use in the third generation suggests that Turkish-origin immigrants still face these issues.

Significant relationships were observed between substance use among Turkish-origin immigrants in Germany and factors such as generation, age, and sex. According to generational analysis, 9.4% of the first generation, 24.9% of the second generation, and 29.4% of the third generation used substances. According to the logistic regression model, the likelihood of substance use among individuals in the third generation was 1.37 times higher compared to the second generation and 2.70 times higher compared to the first generation. The young age group in the third generation also reflected higher usage rates compared to other age groups.

In terms of sex, although not statistically significant, men were 1.12 times more likely to use substances than women (p > 0.05). This aligns with previous research findings that age and sex are risk factors for alcohol and substance use. Salas-Wright et al. provided evidence that substance use among immigrants varies based on age and sex differences [18]. Bucerius examined the changing social and cultural conditions among immigrant groups in Germany over the years, including acculturation, reduced social stress, and generational changes, and concluded that successful acculturation, facilitated by language acquisition, reduces social stress and exclusion but failure in acculturation can increase stress over the years and lead to new and larger risk factors for substance use [19].

Tobacco and alcohol use are considered primary risk factors leading to substance use. According to a study by the Turkish Green Crescent Society, 97% of substance users started with tobacco and 99% with alcohol.^4^ Myers and Kelly noted that smoking and alcohol use often develop concurrently, particularly among youth in treatment for alcohol and other substance use disorders [20]. Vega and Gil found that regular smokers among youth are significantly more at risk for marijuana and other substance use or dependency [21]. These findings are consistent with the results of our study.

Especially for young immigrants, social integration and the overcoming of language barriers are crucial and can reduce both risk factors and substance use itself. It is recommended to implement special projects and policies targeting new generations, particularly the third generation, and to conduct more qualitative and quantitative studies examining the patterns of substance use among young immigrants.

## Figures and Tables

**Table 1:** Dependent and independent variables examined within the scope of the study.

Dependent variable	
Substance use	“I have used it at some point in my life or still use it.” “I have never used.”
Independent variables	
Generation	First generation: Born in Türkiye and migrated to Germany Second generation: Born in Germany with at least one parent born in Türkiye Third generation: Both the individual and his/her parents were born in Germany
Parental consanguinity status	Noted as “Yes” or “No”
Number of individuals over the age of 18 living in the same household	Grouped as 0–2 or ≥3
Tobacco smoking	“I have used it at some point in my life or still use it.” “I have never used.”
Alcoholic beverages	“I have used it at some point in my life or still use it.” “I have never used.”

**Table 2:** Sociodemographic characteristics of Turkish-origin participants living in Germany.

	n	%

Generation (n = 1157)		
First generation	781	67.5
Second generation	359	31.0
Third generation	17	1.5

Age distribution (n = 1157)		
18–19	50	4.3
20–24	122	10.5
25–29	119	10.3
30–34	98	8.5
35–39	115	9.9
40–44	140	12.1
≥45	513	44.3

Sex (n = 1157)		
Male	588	50.8
Female	569	49.2

Parental consanguinity status (n = 1098)		
Yes	155	14.1
No	943	85.9

Years of education (n = 846)		
1–8	209	24.7
9–15	434	51.3
≥16	203	24.0

Occupation over the last 12 months (n = 1118)		
Worker/salaried employee/civil servant	465	41.6
Tradesperson	125	11.2
Student	175	15.7
Housewife	123	11.0
Retired	134	12.0
Unemployed	96	8.6

Number of individuals over the age of 18 living in the same household (n = 1127)		
0–2	632	56.1
≥3	495	43.9

**Table 3:** Tobacco smoking, substance use, and alcoholic beverage consumption status of Turkish-origin participants living in Germany.

	n	%

Tobacco smoking (n = 1157)		
“I have used it at some point in my life or still use it.”	526	45.5
“I have never used.”	631	54.5

Substance use (n = 1132)		
“I have used it at some point in my life or still use it.”	165	14.6
“I have never used.”	967	85.4

Alcoholic beverages (n = 1145)		
“I have used it at some point in my life or still use it.”	362	31.6
“I have never used.”	783	68.4

**Table 4:** Substance use status according to the demographic characteristics of Turkish-origin migrants living in Germany.

Status of using addictive substances	Ever used		Never used		Total		p

	n	%	n	%	n	%	

Generation							<0.001
First generation	71	9.4	687	90.6	758	100.0	
Second generation	89	24.9	268	75.1	357	100.0	
Third generation	5	29.4	12	70.6	17	100.0	

Age group							<0.001
18–19	24	48.0	26	52.0	50	100.0	
20–24	29	24.2	91	75.8	120	100.0	
25–29	31	27.0	84	73.0	115	100.0	
30–34	24	24.7	73	75.3	97	100.0	
35–39	18	16.4	92	83.6	110	100.0	
40–44	28	20.4	109	79.6	137	100.0	
≥45	11	2.2	492	97.8	503	100.0	

Sex							<0.001
Female	52	9.4	503	90.6	555	100.0	
Male	113	19.6	464	80.4	577	100.0	

Parental consanguinity status							0.88
No	141	15.2	786	84.8	927	100.0	
Yes	24	15.7	129	84.3	153	100.0	

Occupation (last 12 months)							<0.001
Worker/salaried employee/civil servant	73	16.0	383	84.0	456	100.0	
Tradesperson	25	20.5	97	79.5	122	100.0	
Student	43	35.8	77	64.2	120	100.0	
Housewife	2	1.2	168	98.8	170	100.0	
Retired	4	3.0	129	97.0	133	100.0	
Unemployed	11	11.7	83	88.3	94	100.0	

Number of individuals over the age of 18 living in the same household							0.523
0–2	88	14.1	535	85.9	623	100.0	
≥3	75	15,5	409	84.5	484	100.0	

Tobacco smoking							<0.001
Never used	46	7.5	570	92.5	616	100.0	
Ever/current user	119	23.1	397	76.9	516	100.0	

Alcoholic beverages							<0.001
Never used	42	5.4	730	94.6	772	100.0	
Ever/current user	122	34.6	231	65.4	353	100.0	

**Table 5:** Logistic regression analysis of risk factors influencing substance use among Turkish-origin immigrants in Germany.

	B	S.E.	Wald	df	p	Exp(B)	95% CI for Exp(B)	
							Lower	Upper

Third generation (ref.)			48.60	2	0.000		1.94	3.95
Second generation	0.45	0.60	15.30	1	0.043	1.37	0.48	3.03
First generation	1.02	0.18	31.30	1	0.018	2.70	0.66	4.29

Sex (male)	0.19	0.36	1.25	1	0.869	1.12	0.53	2.37

Parental consanguinity (yes)	0.19	0.36	1.25	1	0.869	1.12	0.53	2.37

Number of individuals over the age of 18 living in the same household (3 or more)	0.30	0.20	2.25	1	0.134	1.35	0.91	2.01

Tobacco smoking (ever used)	0.94	0.21	19.47	1	0.000	2.55	1.68	3.86

Alcoholic beverages (ever used)	1.90	0.21	80.89	1	0.000	6.67	4.41	10.08

Constant	−2.01	0.65	0.30	1	0.523			

Reference categories are provided in parentheses.

## Footnotes

1 Federal Statistical Office (2023). Press Release No. 080 [online]. Website https://www.destatis.de/EN/Press/2023/03/PE23_080_12.html [accessed 11 January 2024].

2 Federal Statistical Office (2023). Migration and Integration Statistics [online]. Website https://www.destatis.de/EN/Themes/Society-Environment/Population/Migration-Integration/_node.html [accessed 11 January 2024].

3 World Health Organization (2022). Refugee and Migrant Health [online]. Website https://www.who.int/news-room/fact-sheets/detail/refugee-and-migrant-health [accessed 24 September 2023].

3 Turkish Green Crescent Society (2023). Sigara ve alkol bağımlılığı ile madde bağımlılığı arasında nasıl bir bağ vardır? [online]. Website https://www.yesilay.org.tr/tr/sikca-sorulan-sorular/madde-bagimliligi/sigara-ve-alkol-bagimliligi-ile-madde-bagimliligi-arasinda (in Turkish) [accessed 24 September 2023].
